# Body Composition Analysis in Patients with Metabolic Dysfunction-Associated Fatty Liver Disease

**DOI:** 10.3390/nu15183878

**Published:** 2023-09-06

**Authors:** Saori Onishi, Akira Fukuda, Masahiro Matsui, Kosuke Ushiro, Tomohiro Nishikawa, Akira Asai, Soo Ki Kim, Hiroki Nishikawa

**Affiliations:** 1 Second Department of Internal Medicine, Osaka Medical and Pharmaceutical University, Takatsukishi 569-8686, Japan; 2 Health Science Clinic, Osaka Medical and Pharmaceutical University, Takatsuki 569-8686, Japan; 3 Department of Gastroenterology, Kobe Asahi Hospital, Kobe 653-8501, Japan

**Keywords:** MAFLD, fat-free mass, sarcopenia, skeletal muscle mass, BMI

## Abstract

We sought to examine body composition using bioimpedance analysis in patients with metabolic dysfunction-associated fatty liver disease (MAFLD, 2014 males and 949 females). Factors linked to the fat-free mass index (FF index) were examined using univariate and multivariate analysis. An FF index < 18 kg/m^2^ in males and an FF index < 15 kg/m^2^ in females were defined as having decreased skeletal muscle mass. The median age and body mass index (BMI) were 55 years and 25.4 kg/m^2^ in males, and 57 years and 25.4 kg/m^2^ in females, respectively. The FF index strongly correlated with muscle mass index both in males (*r* = 0.999) and females (*r* = 0.999). The prevalence of patients with an FF index < 18 kg/m^2^ in males and an FF index < 15 kg/m^2^ in females was well stratified according to age, BMI, severity of FL, and FIB4 index. In the males, in the multivariate analysis, BMI (*p* < 0.0001), fat mass index (*p* < 0.0001), and waist circumference (*p* = 0.0050) were found to be significant factors linked to FF index. In the females, in the multivariate analysis, BMI (*p* < 0.0001) and fat mass index (*p* < 0.0001) were found to be significant. In conclusion, fat accumulation as reflected by BMI, which is an easily available marker, could be a useful indicator for the skeletal muscle mass in MAFLD.

## 1. Introduction

Determination of low muscle mass for the diagnosis of sarcopenia requires measurement of appendicular skeletal muscle mass (ASMM) using dual-energy x-ray absorptiometry (DEXA) or bioimpedance (BIA), or measurement of skeletal muscle mass using computed tomography (CT), etc. [1,2]. The DEXA method is difficult to transport to medical check-up sites due to the large size and lack of mobility of the measuring equipment, and there are concerns about cost and radiation exposure. Although the BIA method has mobility, relatively low cost, and no concern about radiation exposure, equipment capable of measuring ASMM is not widely available. In addition, it is practically difficult to install CTs in general practice clinics. On the other hand, the fat-free mass index (FFMI, fat-free mass (FFM, kg) divided by the square of height (m)) can be evaluated with body composition analyzers widely available for home use. FFMI has been shown to be a prognostic factor in various diseases [3,4,5,6,7,8]. It has been reported that FFMI correlates well with the ASMM index (ASMMI, ASMM (kg) divided by the square of height (m)) [9]. In the report by Kawakami and colleagues (n = 1313), the percentage of patients with low muscle mass according to the Asian Working Group for Sarcopenia [1] was 5.2% using the BIA method and 9.9% using the DEXA method, and the correlation coefficient between FFMI and ASMMI using the BIA method was 0.96, indicating a strong correlation [9]. They concluded that FFMI can be used as an alternative marker for screening low muscle mass for sarcopenia.

Recently, a new nomenclature for liver disease called metabolic dysfunction- associated fatty liver disease (MAFLD) has been proposed [10]. MAFLD is defined as all patients with fatty liver (FL) and one or more following conditions: (1) overweight or obese (body mass index (BMI) ≥ 23 kg/m^2^ in Japanese patients); (2) type 2 diabetes mellitus; (3) at least two metabolic abnormalities (hypertension, visceral fat accumulation, glucose intolerance, dyslipidemia) in thin or normal weight (BMI < 23 kg/m^2^ in Japanese patients) [10]. MAFLD is characterized by picking up all the FL with metabolic factors that are a risk for cardiovascular events [11,12,13,14]. MAFLD has also been reported to extract cases of advanced liver fibrosis more efficiently than non-alcoholic fatty liver disease (NAFLD) [15].

However, the data for body composition including FFMI in patients with MAFLD are currently scarce compared with NAFLD, while sarcopenia may be a disease modifier in patients with MAFLD [16]. Addressing these issues seems to be clinically meaningful. In this study, we aimed to elucidate the impact of body composition on patients with MAFLD.

## 2. Patients and Methods

### 2.1. Patients

Between February 2022 and May 2023, a total of 2953 consecutive subjects with MAFLD with data for body composition using BIA were identified in our medical records and were retrospectively analyzed in this study. All subjects were examined at the Osaka Medical and Pharmaceutical University (OMPU) Health Sciences Clinic (OMPU-attached facility). All subjects had findings of FL on ultrasonography (US). The severity of FL on US (mild, moderate, and severe) was determined by each examiner. A diagnosis of MAFLD was based on the current guidelines [10]. As mentioned earlier, MAFLD was diagnosed in patients with FL and any of the following (1), (2), or (3): (1) overweight or obese (BMI of 23 kg/m^2^ or higher in Japanese patients), (2) type 2 diabetes mellitus, and (3) BMI less than 23 kg/m^2^, plus two or more metabolic abnormalities of hypertension, visceral fat accumulation, glucose intolerance, or lipid abnormalities [10].

### 2.2. Body Composition Analysis

In the OMPU Health Sciences Clinic, TANITA (body composition analyzer with automatic height meter, DC-270A-N, Tokyo, Japan) has been used for the body composition analysis, and it is a non-invasive measuring instrument. Measurements were taken in the standing and resting positions after obtaining consent for body composition measurement. Total fat mass (kg), total fat-free mass (kg), and total muscle mass (kg) can be measured, but appendicular fat mass, appendicular fat-free mass and appendicular muscle mass cannot be measured in our TANITA. Fat mass index (F index) was defined as fat mass divided by height squared (kg/m^2^). Fat-free mass index (FF index) was defined as fat-free mass divided by height squared (kg/m^2^). Muscle mass index (M index) was defined as muscle mass divided by height squared (kg/m^2^). F index to FF index (F to FF ratio) was defined as F index divided by FF index. Total body muscle mass and total fat-free mass correlate extremely well with ASMM [9]. Based on the previous reports, an FF index < 18 kg/m^2^ in males and an FF index < 15 kg/m^2^ in females were defined as having decreased skeletal muscle mass [9].

### 2.3. Our Study

The percentage of decreased skeletal muscle mass was compared according to baseline characteristics (age, BMI, severity of FL on US, and FIB4 index). Factors linked to FF index were also examined using univariate and multivariate analysis. We obtained ethical approval for the study from the ethics committee of OPMU hospital (approval no. 2023-074), and the protocol strictly observed all regulations of the Declaration of Helsinki. Patient consent was waived due to the retrospective nature of this study.

### 2.4. Statistics

In the two-group comparison (continuous parameters), Student’s *t*-test, the Mann–Whitney *U*-test, or Pearson’s correlation coefficient *r* was used, as applicable. In the multiple-group comparison (continuous parameters), analysis of variance (ANOVA) or the Kruskal–Wallis test was used, as applicable. Factors (continuous variables) with statistical significance for the correlation with FF index were subjected to multivariate regression analysis with multiple predictive variables using the least squares method to select candidate parameters. The normal distribution of residuals was checked as a prerequisite for multiple regression analysis. Unless otherwise mentioned, data are indicated as number or median (range) value. We considered variables of *p* < 0.05 as statistically significant. JMP 17.0.0 software (SAS Institute, Cary, NC, USA) was used to carry out statistical analyses.

## 3. Results

### 3.1. Baseline Characteristics

Baseline characteristics in this study (n = 2014 for males and n = 949 for females) is shown in Table 1. The median (range) age and BMI were 55 years (27–88 years) and 25.4 kg/m^2^ (17.9–48.6 kg/m^2^) in males, and 57 years (25–83 years) and 25.4 kg/m^2^ (17.3–43.9 kg/m^2^) in females, respectively (male vs. female: age (*p* = 0.0052) and BMI (*p* = 0.3847)). In terms of severity of FL, mild, moderate, and severe FL was found in 854 (42.4%), 858 (42.6%), and 302 (15.0%) in male patients, respectively, and 459 (48.3%), 381 (40.2%), and 109 (11.5%) in female patients, respectively (male vs. female: *p* = 0.0029). Regarding waist circumference (WC), one of the diagnostic criteria for metabolic syndrome, a WC of 85 cm or more was found in 78.9% (1590 subjects) of men, and WC of 90 cm or more was found in 44.2% (419 subjects) of women (*p* < 0.0001) [17]. Alanine aminotransferase (ALT) > 30 IU/L was found in 790 subjects (39.2%) in males and 200 subjects (21.1%) in females (*p* < 0.0001).

**Figure 1:**
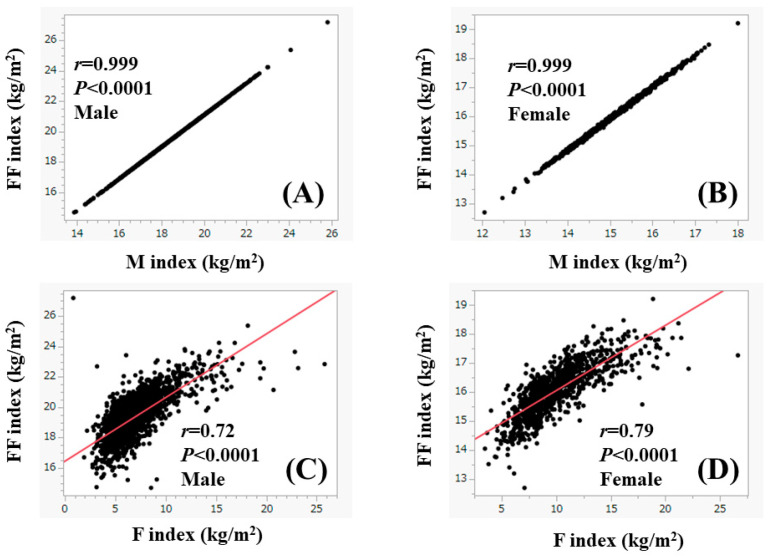
The correlation between FF index (fat-free mass divided by the square of height) and M index (muscle mass divided by the square of height) in males (**A**) and females (**B**). The correlation between FF index and F index (fat mass divided by the square of height) in males (**C**) and females (**D**).

**Figure 2:**
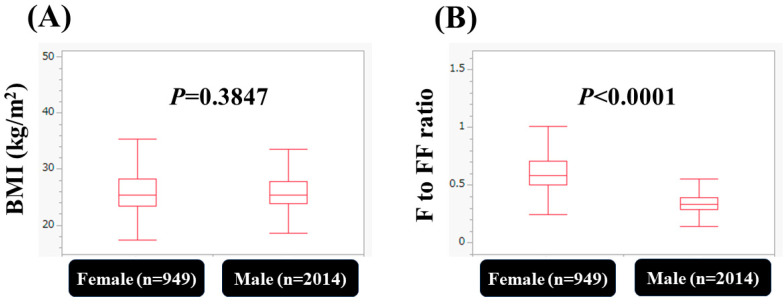
(**A**) Comparison of body mass index between females and males. (**B**) Comparison of F to FF index (fat mass index divided by fat-free mass index) between females (n = 949) and males (n = 2014).

The median (range) F index, FF index, and M index were 6.4 kg/m^2^ (0.84–25.8 kg/m^2^), 19.2 kg/m^2^ (14.7–27.2 kg/m^2^), and 18.2 kg/m^2^ (13.9–25.8 kg/m^2^) in male patients, respectively, and 9.4 kg/m^2^ (3.5–26.7 kg/m^2^), 16.0 kg/m^2^ (12.7–19.2 kg/m^2^) and 15.1 kg/m^2^ (12.1–18.0 kg/m^2^) in female patients, respectively (male vs. female: all *p* < 0.0001). The percentage of FF index < 18 kg/m^2^ in male patients (i.e., skeletal muscle mass loss) was 14.8% (297/2014), while the percentage of FF index < 15 kg/m^2^ in female patients (i.e., skeletal muscle mass loss) was 12.2% (116/949) (*p* = 0.0643). FF index strongly correlated with M index both in males (*r* = 0.999, *p* < 0.0001) and females (*r* = 0.999, *p* < 0.0001) (Figure 1A,B). FF index strongly correlated with F index both in males (*r* = 0.72, *p* < 0.0001) and females (*r* = 0.79, *p* < 0.0001) (Figure 1C,D). In terms of BMI, there was no significant difference between genders (*p* = 0.3847), while in terms of F–FF ratio, there was a significant difference between genders (*p* < 0.0001) (Figure 2A,B).

### 3.2. Percentage of Patients with FF Index Less than 18 kg/m^2^ in Males and FF Index Less than 15 kg/m^2^ in Females According to Age

**Figure 3:**
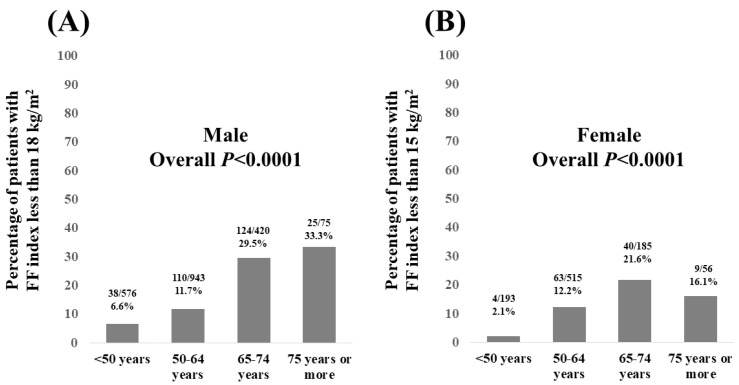
(**A**) Percentage of male patients with FF index less than 18 kg/m^2^ (skeletal muscle mass decrease) according to age. (**B**) Percentage of female patients with FF index less than 15 kg/m^2^ (skeletal muscle mass decrease) according to age.

In males, the percentage of patients with an FF index < 18 kg/m^2^ according to age was 6.6% (38/576) in patients aged <50 years, 11.7% (110/943) in patients aged 50–64 years, 29.5% (124/420) in patients aged 65–74 years, and 33.3% (25/75) in patients aged 75 years or older (overall *p* < 0.0001, Figure 3A).

In females, the percentage of patients with an FF index < 15 kg/m^2^ according to age was 2.0% (4/193) in patients aged <50 years, 12.2% (63/515) in patients aged 50–64 years, 21.6% (40/185) in patients aged 65–74 years, and 16.1% (9/56) in patients aged 75 years or older (overall *p* < 0.0001, Figure 3B).

### 3.3. Percentage of Patients with FF Index Less than 18 kg/m^2^ in Males and FF Index Less than 15 kg/m^2^ in Females According to BMI

**Figure 4:**
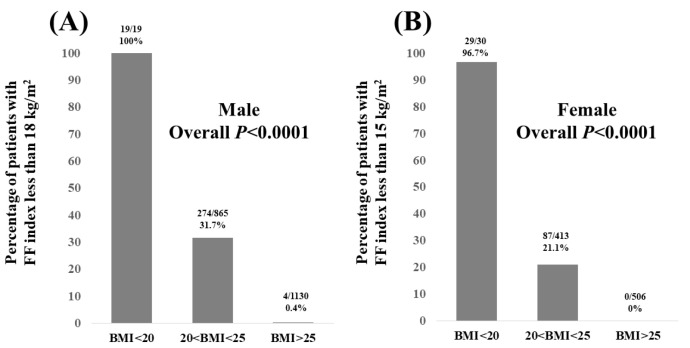
(**A**) Percentage of male patients with FF index less than 18 kg/m^2^ according to BMI. (**B**) Percentage of female patients with FF index less than 15 kg/m^2^ according to BMI.

In males, the percentage of patients with an FF index < 18 kg/m^2^ according to BMI was 100% (19/19) in patients with BMI < 20 kg/m^2^, 31.7% (274/865) in patients with 20 < BMI < 25 kg/m^2^, and 0.4% (4/1130) in patients with BMI > 25 kg/m^2^ (overall *p* < 0.0001, Figure 4A).

In females, the percentage of patients with an FF index < 15 kg/m^2^ according to BMI was 96.7% (29/30) in patients with BMI < 20 kg/m^2^, 21.1% (87/413) in patients with 20 < BMI < 25 kg/m^2^, and 0% (0/506) in patients with BMI > 25 kg/m^2^ (overall *p* < 0.0001, Figure 4B).

### 3.4. Percentage of Patients with FF Index Less than 18 kg/m^2^ in Males and FF Index Less than 15 kg/m^2^ in Females According to the Severity of FL on US

**Figure 5:**
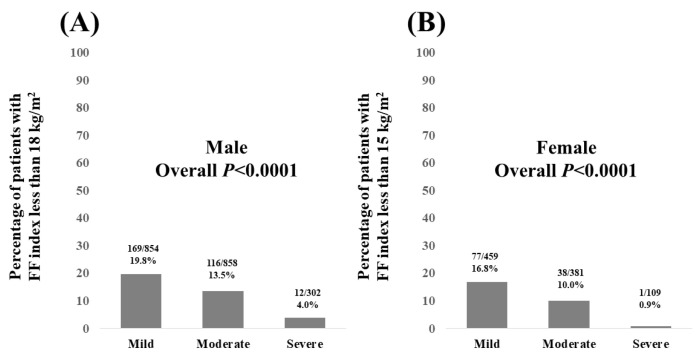
(**A**) Percentage of male patients with FF index less than 18 kg/m^2^ according to the severity of fatty liver. (**B**) Percentage of female patients with FF index less than 15 kg/m^2^ according to the severity of fatty liver.

In males, the percentage of patients with an FF index < 18 kg/m^2^ according to the severity of FL on US was 19.8% (169/854) in patients with mild FL, 13.5% (116/858) in patients with moderate FL, and 4.0% (12/302) in patients with severe FL (overall *p* < 0.0001, Figure 5A).

In females, the percentage of patients with FF index < 15 kg/m^2^ according to the severity of FL on US was 16.8% (77/459) in patients with mild FL, 10.0% (38/381) in patients with moderate FL, and 0.9% (1/109) in patients with severe FL (overall *p* < 0.0001, Figure 5B).

### 3.5. Percentage of Patients with FF Index Less than 18 kg/m^2^ in Males and FF Index Less than 15 kg/m^2^ in Females According to FIB4 Index

**Figure 6:**
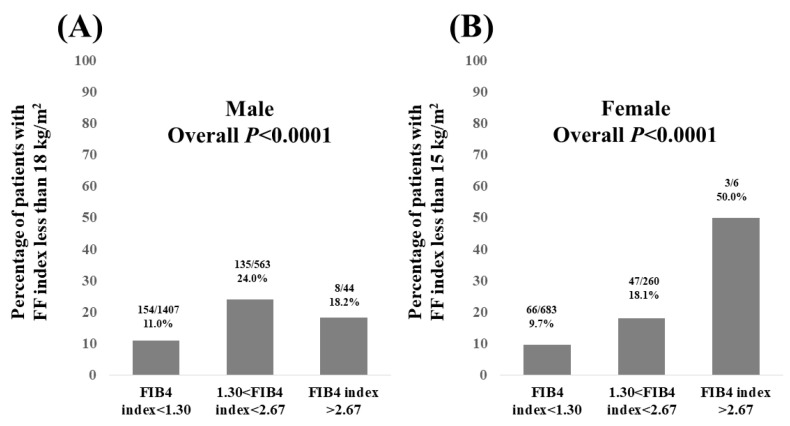
(**A**) Percentage of male patients with FF index less than 18 kg/m^2^ according to the FIB4 index. (**B**) Percentage of female patients with FF index less than 15 kg/m^2^ according to the FIB4 index.

We also analyzed the data according to FIB4 index referring to the current guidelines [18]. In males, the percentage of patients with FF index < 18 kg/m^2^ according to FIB4 index was 11.0% (154/1407) in patients with FIB4 index < 1.30, 24.0% (135/563) in patients with 1.30 < FIB4 index < 2.67, and 18.2% (8/44) in patients with FIB4 index > 2.67 (overall *p* < 0.0001, Figure 6A).

In females, the percentage of patients with FF index < 15 kg/m^2^ according to FIB4 index was 9.7% (66/683) in patients with FIB4 index < 1.30, 18.1% (47/260) in patients with 1.30 < FIB4 index < 2.67, and 50.0% (3/6) in patients with FIB4 index > 2.67 (overall *p* < 0.0001, Figure 6B).

### 3.6. Univariate and Multivariate Analyses of Factors Linked to FF Index

In males, age (*p* < 0.0001), BMI (*p* < 0.0001), WC (*p* < 0.0001), FIB4 index (*p* < 0.0001), F index (*p* < 0.0001), body fat ratio (*p* < 0.0001), and ALT (*p* < 0.0001) were significant factors correlated with FF index (Table 2A). These seven factors were subsequently entered into the multivariate regression analysis. In the multivariate analysis, BMI (*p* < 0.00001), F index (*p* < 0.0001), and WC (*p* = 0.0050) were found to be significant (Table 2B).

In females, age (*p* < 0.0001), BMI (*p* < 0.0001), WC (*p* < 0.0001), FIB4 index (*p* < 0.0001), F index (*p* < 0.0001), platelet count (*p* = 0.0012), body fat ratio (*p* < 0.0001), serum albumin (*p* < 0.0001), and ALT (*p* < 0.0001) were significant factors correlated with FF index (Table 3A). These nine factors were subsequently entered into the multivariate regression analysis. In the multivariate analysis, BMI (*p* < 0.00001) and F index (*p* < 0.0001) were found to be significant (Table 3B).

## 4. Discussion

Sarcopenia, as defined by progressive muscle mass loss and muscle strength decline, has gaining much attention these days due to its prognostic impact, and the number of papers on sarcopenia is rapidly increasing. While MAFLD is a novel disease entity, and its usefulness has been verified from a variety of perspectives, MAFLD is also receiving much research interest worldwide. With the advent of the disease concept of MAFLD, the importance of an approach to metabolic abnormalities became clear [19]. These two clinical entities share a lot of common pathophysiologic mechanisms, and their coexistence may lead to higher rates of morbidity and mortality. Based on these backgrounds, in this study, we examined the body composition in patients with MAFLD, primarily focusing on the FF index. FF index strongly correlated with the M index (*r* = 0.999) both in males and females, and thus a decrease in FF index can be regarded as a decrease in skeletal muscle mass. As shown in Figure 2A,B, despite no significant difference in BMI between men and women, there was a significant difference in the body composition (i.e., F to FF ratio) between men and women. Men have more lean fat mass than women, and women have more fat mass than men, which has been linked to sex hormones [20]. Therefore, we believe that men and women should be discussed separately. As far as we are aware, this study (n = 2963) is one of the largest studies regarding body composition in patients with MAFLD.

In our multivariate analysis, BMI, F index, and WC were found to be significant factors linked to the FF index in males, while in females, BMI and F index were found to be significant factors linked to the FF index. These results denote that BMI and F index can be reliable markers for muscle mass decline in MAFLD patients irrespective of age. Notably, in males, the percentage of patients with FF index < 18 kg/m^2^ was 100% (19/19) in patients with BMI < 20 kg/m^2^, and in females, the percentage of patients with FF index < 15 kg/m^2^ was 96.7% (29/30) in patients with BMI < 20 kg/m^2^. MAFLD patients with BMI < 20 kg/m^2^ should thus receive appropriate interventions. Although not reaching the statistical significance in the multivariate analysis, the aging factor should not be ignored in clinical settings. Aging can be linked to primary sarcopenia and carcinogenesis. MAFLD can be associated with various malignancies [21], while WC was an independent predictor related to FF index decline only in male patients. A possible reason is that there is a closer correlation between WC and BMI in men than in women (*r* = 0.890 in men and *r* = 0.838 in women). WC is an indicator for determining the increase in visceral fat and is included in the mandatory diagnostic criteria for metabolic syndrome [17]. Men are more likely to accumulate visceral fat as a result of obesity than women [22].

In this study, the percentage of BMI < 23 kg/m^2^ and BMI < 25 kg/m^2^ was 11.2% (225/2014) and 42.5% (856/2014) in males, respectively, and 18.8% (178/949) and 44.9% (426/949) in females, respectively. A previous literature review demonstrated that non-obese individuals with FL (i.e., BMI < 25 kg/m^2^) consistently comprised 20% to 35% or more of NAFLD patients, which is in line with our data [23]. Japanese people are more prone to FL than Westerners, even if they are not overweight [18,24]. Approximately 20% of Japanese NAFLD patients have non-obese NAFLD, which has been implicated in the patatin-like phospholipase 3 gene (PNPLA3) [25]. On the other hand, sarcopenic obesity is defined as the co-existence of fat mass increase and sarcopenia and may predict adverse clinical outcomes [26]. Of the USA population, 15.9% had obesity (BMI > 27 kg/m^2^) with low lean muscle mass [27]. In our data, the proportion of male patients with an FF index < 18 kg/m^2^ and BMI > 25 kg/m^2^ was 4.0% (8/2014) and that of female patients with an FF index < 15 kg/m^2^ and BMI > 25 kg/m^2^ was 0% (0/949), which is largely different from the USA data. The reason may be that people who visit the OMPU Health Sciences Clinic are highly concerned about their health and in many cases have appropriate dietary and exercise habits.

The FIB-4 index is an easily available and reliable marker for liver fibrosis in FL patients [28,29]. A higher FIB-4 index can be associated with worse clinical outcomes in NAFLD patients [30]. Liver cirrhosis (LC) can be highly complicated with sarcopenia, and sarcopenia can be an adverse predictor in LC patients [31]. A possible reason why the FIB4 index was not extracted as an independent factor in this study is that the proportion of MAFLD patients with a FIB4 index > 2.67 (i.e., advanced liver fibrosis) is quite low (2.2% (44/2014) in males and 0.6% (6/949) in females).

In our data, ALT > 30 IU/L was found in 790 male subjects (39.2%) and 200 female subjects (21.1%). Recently, the “Nara Statement” has been released from the Japan Society of Hepatology [32]. This declaration pays particular attention to the ALT value, which is widely measured in blood tests as a liver function test in general health examinations and uses ALT over 30 IU/L as an indicator. If a patient has ALT > 30 IU/L in a blood test, he or she should first visit his or her family doctor, who will search for the cause of the disease and, without missing the opportunity, take a full medical examination at a gastroenterologist and receive appropriate medical treatment through cooperation between the family doctor and a specialist. This system should be widely spread.

We acknowledge several limitations in this study. First, this is a single-center cross-sectional observation study with a retrospective nature. Second, the severity of FL on US was determined by each examiner, creating bias. Third, the data for grip strength, which is mandatory for the diagnosis of sarcopenia, are missing in our analysis. The analysis in this study is limited to skeletal muscle mass. Fourth, all medication and alcohol consumption were based on self-report. Thus, data should be carefully interpreted. Nevertheless, our data indicated that fat accumulation as reflected by BMI could be a useful indicator for the skeletal muscle mass in patients with MAFLD.

In conclusion, clinicians should be aware of BMI for the estimation of skeletal muscle mass in patients with MAFLD. Lower BMI can be a risk factor for skeletal muscle decline in patients with MAFLD.

## Figures and Tables

**Table 1:** Baseline characteristics.

	Male (n = 2014)	Female (n = 949)	*p* Value
Age (years)	55 (27–88)	57 (25–83)	0.0052
Body mass index (kg/m^2^)	25.4 (17.9–48.6)	25.4 (17.3–43.9)	0.3847
Waist circumference (cm)	90.5 (70–141)	89 (67–127)	<0.0001
Systolic blood pressure (mmHg)	127 (78–206)	125 (78–188)	0.0756
Diastolic blood pressure (mmHg)	82 (45–131)	78 (43–129)	<0.0001
Anti-hypertension drug, yes/no	694/1320	256/693	0.0002
Severity of FL (mild/moderate/severe)	854/858/302	459/381/109	0.0029
HbA1c (%)	5.8 (4.1–13.9)	5.8 (4.1–10.9)	0.2109
Fasting blood sugar (mg/dL)	97 (67–336)	94 (65–262)	<0.0001
Platelet count (×10^4^/μL)	24.7 (10.6–48.6)	26.9 (13.4–70.1)	<0.0001
Serum albumin (g/dL)	4.4 (3.5–5.2)	4.3 (3.7–5.1)	<0.0001
AST (IU/L)	24 (11–175)	21 (10–176)	<0.0001
ALT (IU/L)	27 (5–307)	20 (5–195)	<0.0001
ALP (IU/L)	68 (22–187)	72 (27–160)	<0.0001
GGT (IU/L)	39 (8–923)	25 (8–612)	<0.0001
FIB4 index	1.03 (0.25–6.73)	1.02 (0.26–3.89)	0.0705
Uric acid (mg/dL)	6.4 (0.8–10.8)	5.3 (2.4–9.9)	<0.0001
Total cholesterol (mg/dL)	207 (90–348)	219 (113–380)	<0.0001
Triglyceride (mg/dL)	127 (24–1681)	108 (27–1226)	<0.0001
Anti-hyperlipidemia drug, yes/no	563/1451	276/673	0.3400
Habitual smoking, yes/no/unknown	430/1515/69	70/828/50	<0.0001
Ethanol intake (g/day)	648/397/397/378/ 125/69	583/187/66/53/ 10/50	<0.0001
0/<20/20–40/40–60/>60/unknown			
HCV antibody, positive/negative	17/1997	8/941	0.9976
HBs antigen, positive/negative	12/2002	1/948	0.0595
Fat-free mass index (kg/m^2^)	19.2 (14.7–27.2)	16.0 (12.7–19.2)	<0.0001
Fat mass index (kg/m^2^)	6.4 (0.84–25.8)	9.4 (3.5–26.7)	<0.0001
Muscle mass index (kg/m^2^)	18.2 (13.9–25.8)	15.1 (12.1–18.0)	<0.0001
F index to FF index ratio	0.344 (0.031–1.129)	0.588 (0.247–1.544)	<0.0001

Data are shown as number or media (range). FL, fatty liver; AST, aspartate aminotransferase; ALT, alanine aminotransferase; ALP, alkaline phosphatase; GGT, γ-glutamyl transpeptidase; HCV, hepatitis C virus; HBs, hepatitis B surface; F index, fat mass index; FF index, fat free mass index.

**Table 2:** Univariate and multivariate analyses of factors linked to FF index in male patients.

(A)			
Univariate	*r*		*p* Value
Age	−0.326		<0.0001
Body mass index	0.882		<0.0001
WC	0.709		<0.0001
Fat mass index	0.719		<0.0001
Body fat ratio	0.592		<0.0001
Platelet count	0.027		0.2265
Serum albumin	0.0137		0.5514
ALT	0.315		<0.0001
ALP	0.0335		0.1385
GGT	0.027		0.2260
FBS	0.039		0.0788
Total cholesterol	−0.0057		0.7987
Triglyceride	0.029		0.1935
FIB4 index	−0.166		<0.0001
**(B)**			
**Multivariate**	**Estimates**	**Standard Error**	***p*** **Value**
Age	9.256 × 10^−5^	8.986 × 10^−5^	0.3031
Body mass index	0.999	0.000905	<0.0001
WC	0.0005014	0.000179	0.0050
Fat mass index	−0.998	0.00229	<0.0001
Body fat ratio	−0.000989	0.000653	0.1303
ALT	−4.653 × 10^−5^	0.000033	0.1598
FIB4 index	−0.000467	0.001634	0.7749

WC, waist circumference; ALT, alanine aminotransferase; ALP, alkaline phosphatase; GGT, γ-glutamyl transpeptidase; FBS, fasting blood sugar.

**Table 3:** Univariate and multivariate analyses of factors linked to FF index in female.

(A)			
Univariate	*r*		*p* Value
Age	−0.359		<0.0001
Body mass index	0.871		<0.0001
WC	0.654		<0.0001
Fat mass index	0.795		<0.0001
Body fat ratio	0.751		<0.0001
Platelet count	0.105		0.0012
Serum albumin	−0.182		<0.0001
ALT	0.216		<0.0001
ALP	−0.011		0.7430
GGT	0.052		0.1068
FBS	0.042		0.1950
Total cholesterol	−0.100		0.0690
Triglyceride	0.065		0.0542
FIB4 index	−0.311		<0.0001
**(B)**			
**Multivariate**	**Estimates**	**Standard Error**	***p*** **Value**
Age	0.0000958	0.000152	0.5295
Body mass index	0.996	0.002105	<0.0001
WC	−0.000233	0.000225	0.3014
Fat mass index	−0.992	0.003502	<0.0001
Body fat ratio	−0.001285	0.000892	0.1501
Platelet count	−0.000413	0.000225	0.0662
Serum albumin	−0.002888	0.004628	0.5328
ALT	−7.068 × 10^−7^	6.523 × 10^−5^	0.9914
FIB4 index	−0.005708	0.004209	0.1754

WC, waist circumference; ALT, alanine aminotransferase; ALP, alkaline phosphatase; GGT, γ-glutamyl transpeptidase; FBS, fasting blood sugar.

## Data Availability

Data available on request due to restrictions eg privacy or ethical.

## Abbreviations

ASMM, appendicular skeletal muscle mass; DEXA, dual-energy X-ray absorptiometry; BIA, bioimpedance; CT, computed tomography; FFM, fat-free mass; FFMI, fat-free mass index; FL, fatty liver; MAFLD, metabolic dysfunction-associated fatty liver disease; NAFLD, non-alcoholic fatty liver disease; BMI, body mass index; OMPU, Osaka Medical and Pharmaceutical University; US, ultrasonography; F index, fat mass index; FF index, fat-free mass index; F to FF ratio, F index to FF index ratio; WC, waist circumference; ALT, alanine aminotransferase; LC, liver cirrhosis.
